# Evaluation of novel computerized tomography scoring systems in human traumatic brain injury: An observational, multicenter study

**DOI:** 10.1371/journal.pmed.1002368

**Published:** 2017-08-03

**Authors:** Eric Peter Thelin, David W. Nelson, Juho Vehviläinen, Harriet Nyström, Riku Kivisaari, Jari Siironen, Mikael Svensson, Markus B. Skrifvars, Bo-Michael Bellander, Rahul Raj

**Affiliations:** 1 Department of Clinical Neuroscience, Karolinska Institutet, Stockholm, Sweden; 2 Division of Neurosurgery, Department of Clinical Neurosciences, University of Cambridge, Cambridge Biomedical Campus, Cambridge, United Kingdom; 3 Section for Perioperative Medicine and Intensive Care, Department of Physiology and Pharmacology, Karolinska Institutet, Stockholm, Sweden; 4 Department of Neurosurgery, University of Helsinki and Helsinki University Hospital, Helsinki, Finland; 5 Department of Neurosurgery, Karolinska University Hospital, Stockholm, Sweden; 6 Department of Anaesthesiology, Intensive Care and Pain Medicine, University of Helsinki and Helsinki University Hospital, Helsinki, Finland; 7 Australian and New Zealand Intensive Care Research Centre, School of Public Health and Preventive Medicine, Monash University, Melbourne, Victoria, Australia; University of Cambridge, UNITED KINGDOM

## Abstract

**Background:**

Traumatic brain injury (TBI) is a major contributor to morbidity and mortality. Computerized tomography (CT) scanning of the brain is essential for diagnostic screening of intracranial injuries in need of neurosurgical intervention, but may also provide information concerning patient prognosis and enable baseline risk stratification in clinical trials. Novel CT scoring systems have been developed to improve current prognostic models, including the Stockholm and Helsinki CT scores, but so far have not been extensively validated. The primary aim of this study was to evaluate the Stockholm and Helsinki CT scores for predicting functional outcome, in comparison with the Rotterdam CT score and Marshall CT classification. The secondary aims were to assess which individual components of the CT scores best predict outcome and what additional prognostic value the CT scoring systems contribute to a clinical prognostic model.

**Methods and findings:**

TBI patients requiring neuro-intensive care and not included in the initial creation of the Stockholm and Helsinki CT scoring systems were retrospectively included from prospectively collected data at the Karolinska University Hospital (*n =* 720 from 1 January 2005 to 31 December 2014) and Helsinki University Hospital (*n =* 395 from 1 January 2013 to 31 December 2014), totaling 1,115 patients. The Marshall CT classification and the Rotterdam, Stockholm, and Helsinki CT scores were assessed using the admission CT scans. Known outcome predictors at admission were acquired (age, pupil responsiveness, admission Glasgow Coma Scale, glucose level, and hemoglobin level) and used in univariate, and multivariable, regression models to predict long-term functional outcome (dichotomizations of the Glasgow Outcome Scale [GOS]). In total, 478 patients (43%) had an unfavorable outcome (GOS 1–3). In the combined cohort, overall prognostic performance was more accurate for the Stockholm CT score (Nagelkerke’s pseudo-*R*^2^ range 0.24–0.28) and the Helsinki CT score (0.18–0.22) than for the Rotterdam CT score (0.13–0.15) and Marshall CT classification (0.03–0.05). Moreover, the Stockholm and Helsinki CT scores added the most independent prognostic value in the presence of other known clinical outcome predictors in TBI (6% and 4%, respectively). The aggregate traumatic subarachnoid hemorrhage (tSAH) component of the Stockholm CT score was the strongest predictor of unfavorable outcome. The main limitations were the retrospective nature of the study, missing patient information, and the varying follow-up time between the centers.

**Conclusions:**

The Stockholm and Helsinki CT scores provide more information on the damage sustained, and give a more accurate outcome prediction, than earlier classification systems. The strong independent predictive value of tSAH may reflect an underrated component of TBI pathophysiology. A change to these newer CT scoring systems may be warranted.

## Abstract 2

Using data from two cohorts, Eric Thelin and colleagues compare the prognostic performance of computerized tomography scoring systems in patients with severe traumatic brain injury.

## Author summary

**Why was this study done?:**

- Most patients who suffer from a significant traumatic brain injury (TBI) undergo head computerized tomography (CT) scanning to visualize injuries.
- The generated image contains information, incorporated into specific “scoring systems,” that can be used to predict patient outcomes and for better stratification of patients in clinical trials.
- Preliminary data have shown that novel CT scoring systems may outperform previous CT scoring systems, but these novel CT scoring systems have not previously been extensively evaluated.

**What did the researchers do and find?:**

- We evaluated 2 novel CT scoring systems (the Stockholm CT score and the Helsinki CT score) in a combined cohort of 1,115 TBI patients from 2 of the larger trauma centers in Europe.
- We found that the 2 novel CT scoring systems systematically outperformed the previous CT scoring systems (the Marshall CT classification and the Rotterdam CT score).
- In addition to other factors known to predict outcome in TBI patients, such as high age and low level of consciousness, the Stockholm and Helsinki CT scoring systems added significant discriminatory performance to outcome prediction systems.
- A more detailed analysis revealed that traumatic subarachnoid hemorrhage was the most important individual component of the CT scores for outcome prediction, presumably due to the lack of effective treatment strategies.

**What do these findings mean?:**

- Our findings suggest that a change to the Stockholm and Helsinki CT scoring systems may be warranted, as they seem to better incorporate clinically relevant injuries.
- Implementation of the novel CT scoring systems could help improve stratification of TBI patients for future clinical trials and also help healthcare providers prioritize resource use.
- We do however acknowledge the need for further validation of these observational findings, preferably through prospective multicenter trials.

## Introduction

Traumatic brain injury (TBI) is one of the most common causes of death among the young [1,2]. Due to changing demographics, it is also an increasing risk factor for morbidity and mortality among the elderly [3]. Upon admission to the hospital, the severity of TBI is commonly graded according to the Glasgow Coma Scale (GCS) [4], a measure of level of consciousness. Although this is of clinical descriptive value, it does not provide any structural information on potential intracranial lesions. Computerized tomography (CT) is the routine imaging modality used to assess structural lesions in acute TBI, due to its accessibility and speed.

The information supplied by the admission CT scan not only allows for diagnostic screening for potential intracranial injuries requiring acute neurosurgical interventions, but also provides important prognostic information. If better implemented, outcome prediction models could help prioritize resources in the emergency setting. Better outcome prediction could also have the potential to improve TBI research by providing baseline risk stratification in trials and to optimize standardization of cohorts in comparative effectiveness research [5].

Currently, several types of CT classification systems exist to prognosticate and stratify TBI patients. Introduced in 1991, the Marshall CT classification [6] categorizes injuries as different levels of diffuse lesions, based on basal cistern compression and midline shift, or focal lesions, depending on whether lesion volume exceeds 25 cm^3^. Despite somewhat arbitrarily chosen cutoffs, this classification is still considered to be somewhat of a “gold standard” for TBI classification. While components of the Marshall CT classification have been shown to contribute to outcome prediction in TBI [7], the Marshall CT classification was not originally designed as a prognostic tool. Thus, in 2005, the Rotterdam CT score was introduced, reweighting components of the Marshall CT classification and adding traumatic subarachnoid hemorrhage (tSAH) and intraventricular hemorrhage [8], creating an ordinal score. Components from the Rotterdam CT score are today an integral part of the International Mission for Prognosis and Analysis of Clinical Trials in TBI (IMPACT) outcome model for TBI patients [7].

More recently, new CT classifications have emerged, including the Stockholm CT score in 2010 [9] and the Helsinki CT score in 2014 [10]. The Stockholm CT score uses midline shift as a continuous variable (as compared to the Marshall CT classification’s and Rotterdam CT score’s threshold of ≥5 mm) and has a separate scoring for tSAH [9]. It is also the only scoring system that takes diffuse axonal injury (DAI) visible on CT into consideration [9]. Moreover, the Stockholm CT score remains the only scoring system that is based on many features of CT scans examined prospectively using an extended protocol, to identify information content. The Helsinki CT score is based on components from both the Marshall CT classification and Rotterdam CT score, but additionally focuses more on the types of intracranial injuries present [10]. Thus, the Stockholm and Helsinki CT scoring systems more comprehensively analyze different components of the admission CT scan, and have both been shown to be better outcome predictors than the Marshall CT classification and Rotterdam CT score [9,10]. However, except for a meeting abstract [11], neither the Stockholm CT score nor the Helsinki CT score has been extensively evaluated, which is crucial in order to determine the generalizability of the scoring systems.

The primary aim of this study was thus to evaluate the Stockholm and Helsinki CT scores for predicting long-term functional outcome using TBI cohorts in both Stockholm and Helsinki, as well as to compare their prediction capabilities with those of the Rotterdam CT score and the Marshall CT classification. Our secondary aims were to examine which components of the Stockholm and Helsinki CT scores best predicted outcome and to determine what independent prognostic value the 2 scoring systems provided in the presence of other IMPACT variables.

## Methods

### Study design and ethics statement

This was an observational database study using prospectively collected data. The study adheres to the STrengthening the Reporting of OBservational studies in Epidemiology (STROBE) statement (S1 Checklist) [12,13], and a study protocol is available (S1 Text). The current study design was approved by the regional ethics committees in both Stockholm (2016/999-31/4) and Helsinki (123/13/03/02/2016 TMK02 § 80). Both committees waived the need for informed consent.

### Study setting

Unconscious TBI patients (GCS of 3–8 at hospital admission) are usually defined as having “severe” TBI [4]. However, as GCS during the first hours following injury is dynamic and has been criticized as not providing adequate assessment of injury severity [14], we chose to create a cohort of patients with “significant” TBI that included TBI patients deemed to be in need of neuro-intensive care unit (NICU) treatment.

Karolinska University Hospital (Stockholm, Sweden) and Töölö Hospital (Helsinki University Hospital, Helsinki, Finland) are the only trauma centers available for patients with TBI requiring NICU care in their regions. They have catchment areas of approximately 2 million people each. TBI patients were included if they were admitted to NICU because of an acute TBI, had prospectively collected long-term outcome data, and had suffered from a blunt TBI (all CT classifications are based on blunt injuries). A flowchart diagram was created to highlight the screening and exclusion of patients, using OmniGraffle (version 7.0, Omni Group, Seattle, Washington, US). Patients in the Karolinska cohort were admitted between 1 January 2005 and 31 December 2014, and patients in the Helsinki cohort were admitted between 1 January 2013 and 31 December 2014. None of the included patients were part of the initial cohorts that were used to create the Stockholm and Helsinki CT scores. Thus, these patients serve as characteristic (more recent patients from the same center) and geographical (patients from another center) evaluation for both scores.

### Treatment

At the NICUs at Karolinska University Hospital and Helsinki University Hospital, we adhered to guidelines similar to those of the Brain Trauma Foundation [15,16]. If mass lesions were present, they were evacuated if deemed appropriate by the attending neurosurgeon. To measure intracranial pressure (ICP), ventricular catheters were predominantly used, even if other pressure devices were sometimes utilized (Codman, DePuy Synthes, Johnson & Johnson, New Brunswick, New Jersey, US, or Rehau AG + Co, Rehau, Germany). The ICP was targeted below the threshold of 20 mm Hg. The head of the patient was elevated at a 30° angle, with the measuring device set at the temple. In case of intracranial hypertension or autonomic dysfunction, cerebral perfusion pressure (CPP) was used to guide treatment, targeted at 50–70 mm Hg, calculated as mean arterial pressure (MAP) minus ICP. CPP control was obtained using vasopressors or intravascular infusions. Unconscious patients were intubated, mechanically ventilated, and sedated with propofol or midazolam in combination with an opiate, either morphine or fentanyl. For patients with refractory high ICP, barbiturate coma was induced (monitored and limited by burst suppression on EEG) or hemicraniectomy was performed. Body temperature was targeted at 36–37°C, regulated predominantly with paracetamol and, if necessary, with parecoxib, ThermoWrap treatment (MTRE Advanced Technologies, Yavne, Israel) or Bair Hugger treatment (3M, Maplewood, Minnesota, US). Microdialysis catheters were inserted to monitor cerebral metabolism (aiming at a lactate:pyruvate ratio < 40) [17], if deemed necessary by the attending neurosurgeon. At Karolinska University Hospital, patients with tSAH were monitored with transcranial Doppler, and if signs of vasospasms were detected, these patients were treated with intravenous infusion of the calcium antagonist nimodipine [18].

### Definition of parameters

Age was used as a continuous variable. Trauma mechanism was similar to the Utstein template, but fewer categories were used [19]. Details on any significant extracranial injury, as defined in the Corticosteroid Randomization after Significant Head Injury (CRASH) trial, were obtained [20]. GCS at admission was used as a continuous variable [21], as previously suggested [20]. Pupil responsiveness was defined as responsive, unilateral unresponsive, or bilateral unresponsive. Intracranial surgery was defined as no surgery (patient was admitted without any intracranial surgery), monitoring surgery (surgery to monitor ICP), evacuation surgery (surgery evacuating traumatic intracranial lesions, returning the bone flap), or hemicraniectomy (decompressive hemicraniectomy by removing the bone flap). Hemoglobin and glucose levels at hospital admission were obtained, if available.

### CT assessment

Marshall CT classification was defined as suggested in previous publications, where grade V (“evacuated mass lesion”) and VI (“non-evacuated mass lesion”) are grouped [8,10]. Rotterdam CT score was classified according to increasing level of severity, as suggested by the authors [8], similarly as the Helsinki CT score [10]. For the Stockholm CT score, the “tally” was used [9]. For details, see Table 1.

**Table 1:** Different CT scoring systems used.

CT classification/scoring system	Classification or component	Description
**Marshall CT classification**	Grade I	No visible intracranial pathology
	Grade II	Midline shift of 0 to 5 mm, basal cisterns remain visible, no high- or mixed-density lesions > 25 cm^3^
	Grade III (swelling)	Midline shift of 0 to 5 mm, basal cisterns compressed or completely effaced, no high- or mixed-density lesions > 25 cm^3^
	Grade IV (shift)	Midline shift > 5 mm, no high- or mixed-density lesions > 25 cm^3^
	Grade V+VI	High- or mixed-density lesions > 25 cm^3^
**Rotterdam CT score**	Basal cisterns	0: normal, 1: compressed, 2: absent
	Midline shift	0: no shift or ≤ 5 mm, 1: shift > 5 mm
	Epidural mass lesion	0: present, 1: absent
	Intraventricular blood or tSAH	0: absent, 1: present
	**Score**	**Sum + 1 (range: 1 to 6)**
**Helsinki CT score**	Mass lesion type, if present	Subdural hematoma: 2, intracerebral hematoma: 2, epidural hematoma: −3
	Mass lesion size	Hematoma volume > 25 cm^3^: 2
	IVH	Present: 3
	Basal cisterns	Normal: 0, compressed: 1, absent: 5
	**Score**	**Sum (range: −3 to 14)**
**Stockholm CT score**	tSAH score	SAH in convexities (1 if 1–5 mm, 2 if >5 mm) + SAH in basal cisterns (1 if 1–5 mm, 2 if >5 mm) + IVH (2 if present) (range: 0–6)
	Tally	Midline shift (mm)/10 + tSAH score/2 − 1 if epidural hemorrhage + 1 if diffuse axonal injury (basal ganglia, splenium, or brain stem) + 1 if dual-sided subdural hematoma + 1

CT, computerized tomography; IVH, intraventricular hemorrhage; SAH, subarachnoid hemorrhage; tSAH, traumatic subarachnoid hemorrhage.

The initial head CT scan after trauma was evaluated in this study to assess all CT scoring systems, which we believe best represents the clinical situation. In the initial article about the Stockholm CT scoring system, the worst CT scan within the first 24 hours after admission to the hospital was used [9], and the Marshall CT classification used subsequent CT scans to determine if mass lesions had been surgically removed [6]. The authors EPT and RR assessed all the CT scans included in this study. EPT assessed the Stockholm CT score, while RR assessed the Helsinki CT score. The Marshall CT classification and the Rotterdam CT score were assessed jointly, and if uncertainties emerged, they were discussed between the 2 authors. To determine inter-examiner variability, both authors assessed the Stockholm and Helsinki CT scores for *n =* 50 scans and found that there was a high degree of concordance (*r* = 0.98 for the Stockholm CT score and *r* = 0.92 for the Helsinki CT score). The examiners were blinded to patient outcome when reviewing CT scans.

### Outcome

At Karolinska University Hospital, patient outcome was determined at 12 months using a structured Glasgow Outcome Scale (GOS) assessment questionnaire or GOS obtained at follow-up appointments [22]. At Helsinki University Hospital, GOS assessments were based on clinical examination and interview by physician 3 to 12 months after TBI. In the analyses, the outcome was dichotomized as an ordinal scale for proportional odds (GOS 1 versus 2 versus 3 versus 4 versus 5), GOS 1–3 versus GOS 4–5 (unfavorable versus favorable outcome), and GOS 1 versus GOS 2–5 (mortality versus survival).

### Statistical analysis

For descriptive purposes, continuous data are presented as medians with interquartile ranges, and categorical data as number and proportion. A univariate regression analysis (“lrm” function in R, “rms” package) [23] was used to correlate different CT and admission variables with different outcome definitions, including a proportional odds model utilizing all steps of GOS and logistic regression towards 2 dichotomizations, unfavorable versus favorable outcome (GOS 1–3 versus GOS 4–5) and mortality versus survival (GOS 1 versus GOS 2–5). The Marshall CT classification and Rotterdam CT score were treated as categorical variables, with the Rotterdam CT score being ordinal [6,8]. The Helsinki CT score was originally constructed as an ordinal scale, but due to its many levels and numeric distribution, it can be treated as a numeric variable [10]. The Stockholm CT score was used as a continuous variable, as suggested by the authors [9]. Summed scores were collapsed to coefficients for each score and patient. In the univariate models, unimputed data were used, thus excluding cases with missing data. Nagelkerke’s pseudo-*R*^2^ and area under the receiver operating characteristic curve (AUC) calculations were used to assess the accuracy of the models, and for comparison with previous studies. Nagelkerke’s pseudo-*R*^2^ gives a value between 0 and 1 resembling explained variance, where 1 indicates a model that fully explains the outcome. In comparison, AUC, with values from 0.5 to 1, is nonlinearly related to Nagelkerke’s pseudo-*R*^2^, with 0.5 indicating at the level of chance and 1 indicating a perfect model. Because most of the CT scores focus on the favorable versus unfavorable outcome dichotomization, this outcome was mainly chosen for the regression models. Differences in performance between models were assessed by testing for significant differences in deviance. Spine plots were used to illustrate how different steps of GOS relate to increasing CT severity scores. Multivariable models including CT parameters and the IMPACT variables age, pupil responsiveness, GCS, and glucose and hemoglobin level (referred to as our “Base model”) [7] were performed to determine the independent outcome information (favorable versus unfavorable outcome) provided by each CT score. In the multivariable regressions, the IMPACT variables’ coefficients were thus reweighted for our population. Unfortunately, the IMPACT variables prehospital hypoxia and hypotension were not available in the Helsinki cohort and were subsequently excluded from the model.

In the original analysis plan, we performed boot-strapping adjustment of the categorical CT scoring systems of the categorical variables. However, as discussed during peer review, this unproportionally penalized the categorical scoring systems. Instead, we used the aforementioned continuous summed scores collapsed to coefficients for each score and patient.

The statistical program R was used (version 3.3.2), utilizing the interface RStudio version 1.0.136 [23]. The statistical significance level was set to *p* < 0.05. The R script used to perform the analyses is available (S2 Text).

### Missing data

Although limited, certain admission data were missing from the digital hospital charts, mainly glucose and hemoglobin levels (see Table 2), and multiple imputation (MI) was performed prior to multivariable analyses, thus utilizing all patients with admission CT and outcome assessments. MI (“mice” package in R) was executed, creating 7 imputed datasets with imputed data drawn from a distribution to retain the uncertainty of the imputed data when ascertaining the significance of predictors. These datasets were then used to create the multivariable models including CT and existing IMPACT variables and their correlations with unfavorable versus favorable outcome. Nagelkerke’s pseudo-*R*^2^ is given as the mean for the 7 imputed datasets. This approach is advocated by the statistical literature as well as the IMPACT research group [24,25] for this type of analysis.

**Table 2:** Patient demographics.

Parameter	Subcategory or units	Karolinska cohort (*n =* 720)	Helsinki cohort (*n =* 395)	Combined cohort (*n =* 1,115)
**Sex**	Male:female	550:170 (76%:24%)	282:113 (72%:28%)	832:283 (75%:25%)
**Age**	Years	52 (32–63)	59 (45–69)	54 (36–65)
**Pre-admission**				
Trauma mechanism	Fall same level	248 (34%)	220 (56%)	468 (42%)
	Fall from a height	180 (35%)	44 (11%)	224 (20%)
	Traffic accident	173 (24%)	60 (15%)	233 (21%)
	Assault	77 (11%)	22 (6%)	99 (9%)
	Other	38 (5%)	9 (2%)	47 (4%)
	Missing	4 (0%)	40 (10%)	44 (4%)
Significant extracranial injury	Present	222 (31%)	56 (14%)	278 (25%)
	Missing	1 (0%)	1 (0%)	2 (0%)
**Admission**				
Glasgow Coma Scale	3–8	440 (61%)	145 (37%)	585 (52%)
	9–13	173 (24%)	98 (25%)	271 (24%)
	14–15	107 (15%)	151 (38%)	258 (23%)
Pupil responsiveness	Responsive	556 (77%)	319 (80%)	875 (78%)
	Unilateral unresponsive	59 (8%)	50 (13%)	109 (10%)
	Bilateral unresponsive	79 (11%)	19 (5%)	98 (9%)
	Missing	26 (4%)	7 (2%)	33 (3%)
Intracranial surgery	No surgery	168 (23%)	171 (43%)	339 (30%)
	Monitoring surgery	205 (28%)	31 (8%)	236 (21%)
	Evacuation surgery	325 (45%)	184 (47%)	509 (46%)
	Hemicraniectomy	21 (5%)	9 (2%)	30 (3%)
Hemoglobin (g/l)	Grams/liter	136 (123–147)	130 (118–140)	133 (121–144)
	Missing	153 (21%)	1 (0%)	154 (14%)
Glucose (mmol/l)	Millimoles/liter	7.9 (6.8–9.8)	8.0 (6.4–9.2)	7.9 (6.6–9.5)
	Missing	309 (43%)	21 (5%)	330 (30%)
**Radiology**				
Marshall CT classification	I	0 (0%)	1 (0%)	1 (0%)
	II	195 (27%)	84 (21%)	279 (25%)
	III	130 (18%)	57 (14%)	187 (17%)
	IV	25 (3%)	6 (2%)	31 (3%)
	V+VI	370 (51%)	247 (63%)	617 (55%)
Rotterdam CT score	1	14 (2%)	10 (3%)	24 (2%)
	2	62 (9%)	37 (9%)	99 (9%)
	3	221 (31%)	107 (27%)	328 (29%)
	4	239 (33%)	113 (29%)	352 (32%)
	5	145 (20%)	104 (26%)	249 (22%)
	6	39 (5%)	24 (6%)	63 (6%)
Stockholm CT score		2.2 (1.5–3.0)	2.3 (1.5–3.0)	2.2 (1.5–3.0)
Helsinki CT score		5 (3–7)	5 (3–7)	5 (3–7)
**NICU**				
NICU stay	Days	6 (2–15)	3 (1–6)	4 (2–11)
**Outcome**				
Time to GOS assessment (survivors)	Days	367 (294–396)	147 (98–214)	320 (132–380)
Long-term GOS	1 (death)	120 (17%)	90 (23%)	210 (19%)
	2 (vegetative state)	6 (1%)	3 (1%)	9 (1%)
	3 (severe disability)	196 (27%)	63 (16%)	259 (23%)
	4 (moderate disability)	233 (32%)	119 (30%)	352 (32%)
	5 (good recovery)	165 (23%)	120 (30%)	285 (26%)
	4–5 (favorable outcome)	398 (55%)	239 (61%)	637 (57%)

Data are presented as n (percent) or median (interquartile range).

CT, computerized tomography; GOS, Glasgow Outcome Scale; NICU, neuro-intensive care unit.

## Results

### Patient demographics

**Fig 1:**
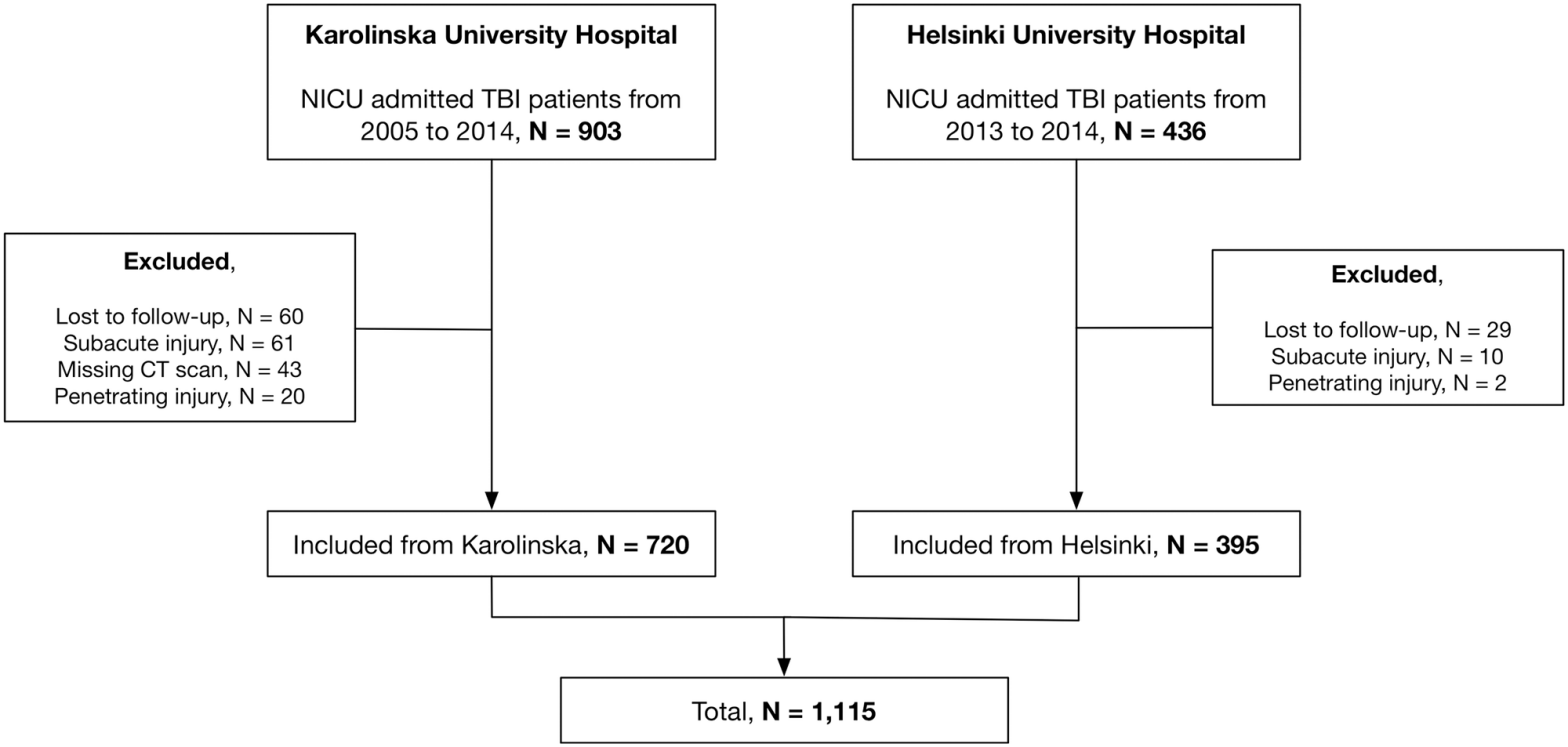
Patient flow diagram. Flowchart diagram of the eligible patients and screening process to exclude patients who did not fulfill inclusion criteria. CT, computerized tomography; NICU, neuro-intensive care unit; TBI, traumatic brain surgery.

A total of 1,115 patients with significant TBI were included from both centers, with a majority from Karolinska University Hospital in Stockholm, Sweden (*n* = 720, 65%). A flowchart visualizes the inclusion process (Fig 1). Patients in the Helsinki cohort were slightly older and had more same-level falls and fewer traffic accidents than those in the Stockholm cohort, which presumably explains the higher prevalence of extracranial injuries in the Stockholm cohort (Table 2). According to the GCS, there were more patients with mild TBI (GCS 14–15) and fewer patients with severe TBI (GCS 3–8) in the Helsinki cohort as compared to the Stockholm cohort, but with a similar degree of pupil responsiveness. More patients in the Stockholm cohort underwent monitoring surgery (28% versus 8%), while near half of the patients in the Helsinki cohort did not have any intracranial surgery performed at all (43%, as compared to 23% in the Stockholm cohort) (Table 2).

Radiographically, the Helsinki cohort had more patients with Marshall Grade V+VI (focal mass lesions > 25 cm^3^, 63% versus 51%), but the 2 cohorts had similar intracranial severity according to the Helsinki and Stockholm CT scoring systems, with a somewhat higher proportion of more severely injured patients according to Rotterdam CT scoring (Table 2). A more detailed description of the distribution of intracranial injuries between the cohorts is presented (S1 Table).

While more patients died in the Helsinki cohort (23% versus 17%), this cohort had fewer patients with GOS 3 (“severe disability”, dependent state) than the Stockholm cohort (16% versus 27%), and more favorable outcomes (GOS 4–5, 61% versus 55%) (Table 2).

### Outcome prediction of CT scores

**Fig 2:**
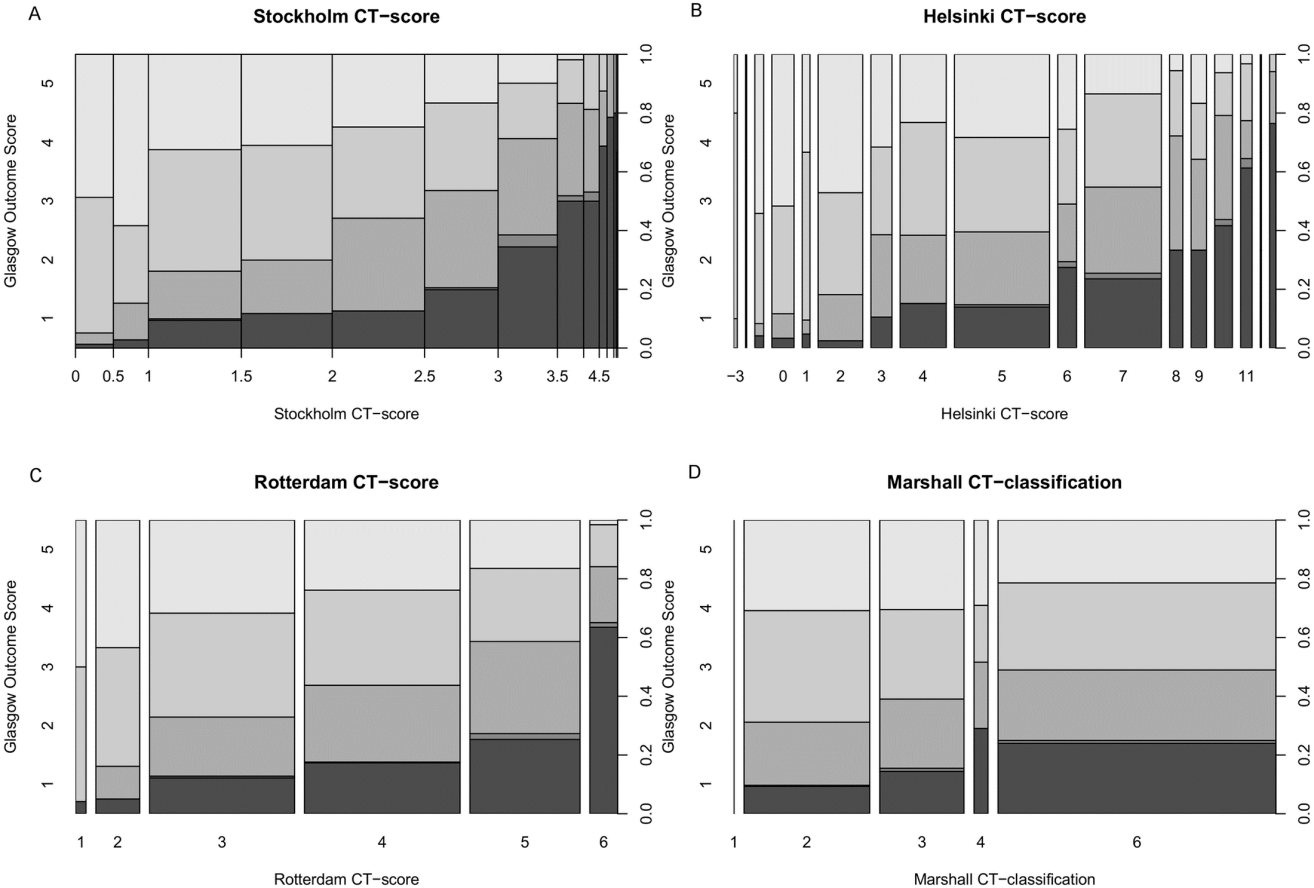
Different CT scores versus outcome. Spine plots were used to illustrate how different levels of GOS relate to an increasing CT severity score for the Stockholm (A), Helsinki (B), and Rotterdam (C) CT scores and the Marshall CT classification (D). Glasgow Outcome Scale (y-axis, left), the CT score (x-axis), and outcome proportions summing to 1 (y-axis, right) are given for all panels. The sizes of the bins correspond to the number of patients in each category. CT, computerized tomography.

The Stockholm and Helsinki CT scores outperformed the Rotterdam CT score and Marshall CT classification in the combined patient cohort in all outcome dichotomizations. The Stockholm CT score was marginally more accurate in all models, reaching pseudo-*R*^2^ values as high as >0.30 in some models (Table 3). Generally, the Stockholm and Helsinki CT scores exhibited a pseudo-*R*^2^ in the range of 0.20–0.25 for all outcome models, while the Rotterdam CT score exhibited a pseudo-*R*^2^ of 0.10–0.20, and the Marshall CT classification generally around 0.05 (Table 3). Interestingly, the Helsinki cohort had higher pseudo-*R*^2^ for all CT scoring systems and, thus, a stronger correlation between intracranial pathology and scores, compared to the Stockholm cohort (Table 3). The AUCs yielded, as expected, similar results as Nagelkerke’s pseudo-*R*^2^ (Table 3). The GOS values at different CT score levels are visualized with spine plots (Fig 2). The Stockholm and Helsinki CT scores visually discriminate both GOS outcome dichotomizations well, but principally so the favorable/unfavorable dichotomization they were weighted for (Fig 2A and 2B). The Rotterdam CT score is clearly seen to be ordinal (Fig 2C). The Marshall CT classification is not ordinal, with Grade IV as the worst intracranial state (highest mortality rate) (Fig 2D).

**Table 3:** Validation of the CT scores in the different cohorts.

CT classification/scoring system	Stockholm cohort (*n =* 720)		Helsinki cohort (*n =* 395)		Combined cohort (*n =* 1,115)	
	Pseudo-*R*^2^	AUC (95% CI)	Pseudo-*R*^2^	AUC (95% CI)	Pseudo-*R*^2^	AUC (95% CI)
**Proportional odds (GOS 1 versus 2 versus 3 versus 4 versus 5)**						
Stockholm CT	0.23	NA	0.30	NA	0.26	NA
Helsinki CT	0.10	NA	0.18	NA	0.18	NA
Rotterdam CT	0.09	NA	0.22	NA	0.13	NA
Marshall CT	0.02	NA	0.08	NA	0.03	NA
**GOS 1–3 versus 4–5 (unfavorable versus favorable)**						
Stockholm CT	0.25	0.75 (0.71–0.79)	0.35	0.80 (0.75–0.84)	0.28	0.77 (0.74–0.79)
Helsinki CT	0.21	0.71 (0.67–0.74)	0.30	0.75 (0.71–0.80)	0.22	0.72 (0.69–0.75)
Rotterdam CT	0.12	0.66 (0.62–0.70)	0.25	0.73 (0.68–0.78)	0.15	0.68 (0.65–0.71)
Marshall CT	0.02	0.56 (0.52–0.60)	0.10	0.63 (0.57–0.68)	0.03	0.58 (0.55–0.61)
**GOS 1 versus 2–5 (dead versus alive)**						
Stockholm CT	0.21	0.76 (0.71–0.81)	0.27	0.78 (0.73–0.84)	0.24	0.77 (0.73–0.80)
Helsinki CT	0.18	0.74 (0.69–0.79)	0.26	0.73 (0.67–0.79)	0.19	0.74 (0.70–0.77)
Rotterdam CT	0.09	0.66 (0.61–0.71)	0.22	0.72 (0.65–0.78)	0.13	0.68 (0.64–0.72)
Marshall CT	0.03	0.59 (0.53–0.64)	0.09	0.63 (0.57–0.69)	0.05	0.61 (0.57–0.65)

Performance of CT sores by center, combined, and by outcome dichotomizations. Data are presented as Nagelkerke’s pseudo-R^2^ and AUC comparison between the CT scores.

AUC, area under the receiver operating characteristic curve; CI, confidence interval; CT, computerized tomography; GOS, Glasgow Outcome Scale; NA, not available.

### Different components of the CT scores versus outcome

The tSAH score of the Stockholm CT score was the individual CT component most highly correlated with outcome in all TBI populations (S2 Table), with a univariate Nagelkerke’s pseudo-*R*^2^ of 0.12 in the combined cohort. Moreover, compression of cisterns, the presence of intraventricular hematoma, and the presence of epidural hematoma also presented high pseudo-*R*^2^ values in the models (S2 Table). A notable difference between the cohorts was the impact of midline shift: the Stockholm CT score exhibited a pseudo-*R*^2^ of 0.04 in the Stockholm cohort but 0.20 in the Helsinki patients (S2 Table).

The additional IMPACT variables age, admission GCS, and pupil responsiveness all presented high values of pseudo-*R*^2^ (>0.10). Notably, age and glucose level were better outcome predictors in the Helsinki cohort than in the Stockholm cohort (S2 Table).

### Outcome prediction of CT scores and other parameters

Our Base model, consisting of age, pupil responsiveness, GCS, and hemoglobin and glucose level at hospital admission, displayed an adjusted pseudo-*R*^2^ of 0.38 for favorable versus unfavorable outcome (Table 4). If the Marshall CT classification was added, no independent or significant increase in discriminatory performance was noted for the outcome prediction model. However, if the Rotterdam, Helsinki, or Stockholm CT score was added to the Base model, the adjusted pseudo-*R*^2^ increased to 0.40, 0.42, and 0.44, respectively (Table 4). Thus, the Helsinki and Stockholm CT scores contributed 4% and 6% of additional explained variance, respectively, in the presence of IMPACT variables that are known outcome predictors.

**Table 4:** CT models in multivariable analysis together with available IMPACT variables.

Model	Nagelkerke’s pseudo-*R*^2^
Base model	0.38
Base + Marshall CT	0.39 (*p* = 0.34)
Base + Rotterdam CT	0.40 (*p* < 0.01)
Base + Helsinki CT	0.42 (*p <* 0.01)
Base + Stockholm CT	0.44 (*p <* 0.01)

Base model consists of age, pupil responsiveness, Glasgow Coma Scale, and hemoglobin and glucose level at hospital admission. Nagelkerke’s pseudo-R^2^ values are from multivariable regression models, where a value of 1 would fully predict unfavorable versus favorable outcome (GOS 1–3 versus 4–5). p-Values in parentheses describe whether the CT score significantly added independent information to the model.

CT, computerized tomography; IMPACT, International Mission for Prognosis and Analysis of Clinical Trials in TBI.

## Discussion

To our knowledge, this represents the first published extensive evaluation of the Stockholm and Helsinki CT scoring systems. This study clearly indicates that these novel CT scores, which take into account additional information from the initial CT scan, are superior to the currently widely used CT scoring systems—the Rotterdam CT score and the Marshal CT classification—with the Stockholm CT score being marginally more accurate than the Helsinki CT score. We showed that both the Stockholm and Helsinki CT scores account for more of the pseudo-explained variance in univariate outcome prediction, more than both other CT scores and any single of the other parameters assessed. However, much of the CT information gained correlates with other predictors of TBI, and the increase in information with the addition of a CT score to composite outcome models is—although significant (except for the Marshall CT classification)—less pronounced than in univariate models. Overall, the Stockholm and Helsinki CT scores add independent information to outcome prediction models including IMPACT variables, to an extent that may motivate a switch to their general use.

The Stockholm CT score was found to be the most accurate outcome predictor of the ones tested in this study. At best, it yielded a pseudo-*R*^2^ of 0.35 (in the Helsinki cohort), which is similar to the results achieved in the original development cohort [9]. This is despite the fact that the current study used the initial CT scan and not the “worst” CT scan of the first 24 hours, as was done when the model was created. Using the worst CT scan would be expected to result in more accurate outcome prediction, as it would capture any potentially detrimental lesion progression [26]. Future studies are needed to determine at which time point the head CT provides the most prognostic information.

Interestingly, the Stockholm CT score performed better in the Helsinki cohort. This may be related to differences in patient and injury characteristics, i.e., patients in the Helsinki cohort were older and had higher GCS scores. Additionally, a contributing factor in the Stockholm cohort may be that the knowledge gained from the Stockholm CT score, particularly the impact of midline shift, may have contributed to a local change in practice towards a more aggressive surgical approach, reflected by the higher incidence of surgery for both monitor insertion and hematoma evacuation in the Stockholm cohort. This suggests a possible interesting dynamic interplay between scoring systems and treatment strategies, implying that prediction models could require a more continuous weighting of variables in the future. In addition, the current cohort is a distinctly older population than the patient groups in which the Stockholm CT score has previously been used. A recent conference abstract presenting a study of 48 TBI patients constitutes the only other evaluation of the Stockholm CT score’s prognostic capabilities to date. The authors found an AUC of 0.76 in relation to favorable/unfavorable outcome [11]. The Helsinki CT score, based on 869 NICU-treated TBI patients admitted between 2009 and 2012 to Helsinki University Hospital [10], was more recently published and has not previously been validated. The AUC (0.75) and pseudo-*R*^2^ (0.25) scores for outcome prediction in the Stockholm patient cohort in the current study were similar to what was found in the original Helsinki CT score article, albeit the former were systematically less accurate.

The predictive capabilities of the Rotterdam CT score, modeled using 2,249 patients with moderate-to-severe TBI from a multicenter randomized clinical trial studying the effect of the drug tirilazad (recruiting patients between 1991 and 1994) [27] also exhibited similar AUC (0.68–0.76) and pseudo-*R*^2^ (0.09–0.25) values compared to previous studies [9,10], and discriminating both outcome dichotomizations. However, the Rotterdam CT score systematically resulted in lower outcome prediction discriminatory performance than the Stockholm and Helsinki CT scores in our study. The Marshall CT classification, constructed using the Traumatic Coma Data Bank (TCDB) from 1984 to 1987, and including 746 patients with severe TBI (GCS 3–8), resulted in the lowest explained pseudo-variance in comparison to the other scoring systems and did not yield any independent information if added to admission characteristics. While previous studies have found lower explained pseudo-variance values for the Marshall CT classification in outcome predictions, as compared to the Rotterdam CT score [9,28], the pseudo-variance values have not been as low as seen in this study. We have no immediate explanation for this, but given that TBI populations, surgical and NICU management, and the general quality of databases may have changed since the mid-1980’s, there are several potential explanations for why the Marshall CT classification may provide less information today. Notably, the Marshall CT classification was never meant to be used for outcome prediction as it is not an ordinal score (the authors acknowledge that Grade IV is worse than Grade V and VI) [6]. Moreover, the Marshall CT classification is limited in that it neither takes SAH into account nor discriminates between epidural and subdural hematoma. Furthermore, the somewhat arbitrary cutoff of >25 cm^3^ for a “mass lesion” leads almost all extra-parenchymal bleedings to be classified as Marshall Grade VI. This produces a problematic distribution of patients between categories in current populations, and decreases the granularity of the scoring system. Another limitation that has previously been acknowledged [29] is the existence of the “evacuated mass lesion”/Grade V category, making the Marshall CT classification difficult to compare to the other CT scores, as they only evaluate pre-operative CT scans. This was the motivation for fusing Marshall Grade V and Grade VI into a “mass lesion” group. In summary, the Rotterdam CT score and Marshall CT classification underperformed in the current study, presumably due to their inclusion of fewer, and today less clinically relevant, intracranial parameters.

The component analysis revealed that the tSAH score of the Stockholm CT score was the strongest unique outcome predictor. In the 3 CT scores with subcomponents, the tSAH variable is seen to be an important outcome predictor. However, the Stockholm CT score discriminates more levels of tSAH than the Helsinki CT score (presence of IVH) or Rotterdam CT score (presence of IVH/tSAH), while tSAH/IVH is not part of the Marshall CT classification. Diffuse bleeding stemming from subarachnoid vessels in TBI is a well-known predictor of unfavorable outcome [30,31]. It has been shown that tSAH in TBI patients can, similarly to aneurysmal SAH, induce vasospasm and ischemia [32], potentially triggering harmful inflammatory and neurotoxic processes, which are also potential targets for several neuroprotective drugs [33]. Despite this, a key finding in this study is that the degree of tSAH is an independent outcome predictor in TBI, suggesting that pathophysiological processes related to tSAH are of greater importance in TBI than generally considered.

While CT-visible DAI on the admission scan has been shown to be associated with an unfavorable outcome [34], DAI findings did not correlate significantly with outcome in this study. Our findings are, however, in line with the original Stockholm CT score article, in which no type of DAI on CT was significantly correlated to an unfavorable outcome in the univariate analysis, but more central DAI provided significant information in multivariable models [9]. In the original Stockholm CT score article, the DAI component contributed little to discriminatory performance, probably due to the low incidence of DAI, but was found to enhance the calibration of models. Additionally, as the populations in the current study comprise a slightly older patient cohort with a generally lower prevalence of DAI (perhaps due to a lower incidence of high-energy trauma [9] than used to originally weigh and create the score), it is possible that the predictive role of DAI is different in this cohort. Overall, future revision of variables using both the Helsinki and Stockholm CT scores may provide cause for altering both weighting and variables in a future composite score, including the DAI variable.

Mass effect indicators, such as midline shift and lesions larger than 25 cm^3^, exhibited low predictive value in this study, especially in the Stockholm cohort. This could be indicative of a trend where mass lesions are not as deleterious as they once were, due to improved pre-hospital management, rapid imaging, and neurosurgical hematoma evacuation [20,35]. Longer periods from the time of injury to surgical evacuation may have negative effects on patients with intracranial-space-occupying lesions. However, a recent review suggests this to be debatable [36]. A more conservative approach to neurosurgical interventions for intracranial mass lesions in other studied cohorts has not been consistently related to worse outcome [37], supporting that patients are arguably better treated today than 20 years ago, including conservative medical treatments, if adequately monitored. Midline shift was a strong predictor in the Helsinki cohort, most likely related to the strong association between age and outcome, especially in patients with subdural hematoma [38]. Overall, mass lesion parameters provided less predictive outcome information than previously, presumably as a result of general improvement of the healthcare system.

The Stockholm CT score, whilst being more accurate than Helsinki CT score, could be considered more complex as it includes CT-visible DAI and SAH grading, which requires a more trained CT examiner. In comparison, the Helsinki CT score is easier and faster in its approach, even if the CT examiners occasionally found it difficult to determine whether “intracerebral hematoma/contusions” were present in the parenchyma or in the subarachnoid space. There are also subjective issues specific to the Rotterdam and Helsinki CT scores, such as interpretations of “compressed” versus “obliterated” basal cisterns, as well as when mass lesions are >25 cm^3^ as the “ABC/2” method is only an estimate [39]. However, the inter-examiner analysis supported that, despite these more subjective characteristics, there was a high congruence of results. In summary, while the Helsinki CT score is easier to assess than the Stockholm CT score, it still contains some subjective interpretation, which can affect scoring between centers and examiners.

There are several limitations in this study that should be acknowledged. This is a retrospective analysis of prospectively collected data in predefined databases, and variables that cannot be matched between centers are less retrievable. While information on comorbidities, which could in part shed light on differences between sites, was available in the Helsinki cohort, it was not in the Stockholm cohort. Moreover, the Helsinki cohort lacked pre-hospital hypoxia and hypotension data (parts of the IMPACT model “Core+CT”), which were available in the Stockholm cohort but not presented in the study. Additionally, the Stockholm cohort had a relatively high incidence of missing admission glucose level, due to changes over time in the digitalization of emergency charts. However, we do not believe that this constitutes a major limitation as admission glucose (and hemoglobin) level was of marginal importance in the prediction models, and MI was performed [24].

As designed, this study constitutes a type of external validation of the CT scores (versus outcome) with characteristic (more recent patients from the same center) and geographic (patients from another center) external validation cohorts [40]. We did not, however, perform an internal validation, which would also include evaluating calibration of the CT scores [41]. As we instead used the summed scores of all 3 scoring systems, we in effect evaluated the extent to which information content could be discriminated between scores. New reweightings and assessment of model calibration in contemporary populations should be the scope of future studies. Moreover, as the CT scores were validated in the same centers by the same authors behind the original studies, this could be considered a source of bias, and the type of external validation could be considered “weak” [42,43]. To some extent this was addressed by blinding the CT assessors to patient outcomes. However, both the Helsinki and Stockholm CT scores require further external validation and possibly new weightings of variables from other studies, such as the upcoming CENTER-TBI [44].

The time to GOS outcome assessment differed between the 2 centers, averaging close to 1 year at Karolinska University Hospital and about 6 months at Helsinki University Hospital. TBI patients have been shown to improve over time, suggesting that a later time point would yield improved assessments [45]; thus, we potentially underestimated the outcomes for the Helsinki cohort. In our experience, and supported by the literature [45,46], the patients who primarily improve over time in NICU cohorts are GOS 3 patients becoming GOS 4 or better, and to a lesser extent GOS 4 patients becoming GOS 5. Because of the dichotomizations of GOS used in the analyses, GOS 3 patients becoming GOS 4 (crossovers) would potentially cause the greatest bias. However, inspecting the Helsinki cohort, there were relatively few GOS 3 patients (16%), making it unlikely that we would have seen major differences in outcome in this group if assessed at 1 year, and thus crossovers presumably do not constitute a major limitation.

Finally, in contrast to many TBI studies, we included all NICU-treated TBI patients, thus mixing patients traditionally classified as having “mild,” “moderate,” and “severe” TBI based on the admission GCS. However, GCS definitions of injury severity are under scrutiny for several reasons. GCS is an uncertain discriminator as it is influenced by a multitude of factors including drugs and sedative medication [47], its subjective nature [48], and its dynamic behavior during the first day [49]. We believe that a cohort consisting of TBI patients deemed to be in need of intensive care represents a clinically valid group of patients with significant TBI. In an exploratory subgroup analysis, we examined patients with an admission GCS of 3–8 (*n =* 586) and found that the Stockholm CT score had a pseudo-*R*^2^ of 0.28, Helsinki CT score, 0.25, Rotterdam CT score, 0.16, and Marshall CT classification, 0.08, in GOS 1–3 versus GOS 4–5 dichotomized models; thus, the results were similar to those of the complete patient cohort. Overall, the considered limitations we present are in our estimation minor and do not diminish the main conclusions of this study.

### Conclusion

In this extensive external validation study, we found that the Stockholm and Helsinki CT scores were more accurate outcome predictors after TBI than the Rotterdam CT score or the Marshall CT classification. A switch to granular CT scoring systems may be warranted. Specifically, much of the additional information provided by the Stockholm CT score is derived from a more differentiated description of tSAH, suggesting that the amount and location of tSAH plays a larger role in TBI outcome than previously assumed and could open new therapeutic windows in TBI. In this study, we focused on and compared the information content of the summed CT score components, and not the given weightings to produce predicted probabilities. CT scoring systems will need to be reweighted over time to adjust for changes in demographics and treatments affecting the importance of predictor variables.

## Supporting information

S1 Checklist STROBE checklist. STrengthening the Reporting of OBservational studies in Epidemiology (STROBE) checklist for the current study.
(DOC)

S1 Table Prevalence of CT score components between centers. (DOCX)

S2 Table Pseudo-explained variance (Nagelkerke’s pseudo-*R*^2^) for the components of the Base model and CT score models. (DOCX)

S1 Text Study protocol. The study protocol used when designing the study.
(DOCX)

S2 Text R script. The code used for the statistical models in the study.
(R) Click here for additional data file.

## Abbreviations

AUC: area under the receiver operating characteristic curve
CT: computerized tomography
DAI: diffuse axonal injury
GCS: Glasgow Coma Scale
GOS: Glasgow Outcome Scale
ICP: intracranial pressure
IMPACT: International Mission for Prognosis and Analysis of Clinical Trials in TBI
IVH: intraventricular hemorrhage
MI: multiple imputation
NICU: neuro-intensive care unit
SAH: subarachnoid hemorrhage
TBI: traumatic brain injury
tSAH: traumatic subarachnoid hemorrhage
