# Correction: Evaluation of novel computerized tomography scoring systems in human traumatic brain injury: An observational, multicenter study

**DOI:** 10.1371/journal.pmed.1004320

**Published:** 2023-11-28

**Authors:** The PLOS Medicine Staff

## Notice of republication

This article was republished on November 9, 2023, to remove a Supporting Information file that was incorrectly included in the originally published article. Please download this article again to view the correct version.Reference
