# Supplementary material for: Evaluation of novel computerized tomography scoring systems in human traumatic brain injury: An observational, multicenter study
Source: PLoS Med. 2017 Aug 3;14(8):e1002368. doi: 10.1371/journal.pmed.1002368 (PMC5542385; doi:10.1371/journal.pmed.1002368)
Supplement: S1 Table — (DOCX) [file pmed.1002368.s002.docx]

|  | Stockholm (n=720) | Helsinki (n=395) | Combined  (n=1115) |
| --- | --- | --- | --- |
| **Helsinki CT components** |  |  |  |
| ASDH | 540 (75%) | 316 (80%) | 856 (77%) |
| ICH/Contusions | 494 (69%) | 241 (61%) | 735 (66%) |
| EDH | 104 (14%) | 44 (11%) | 148 (13%) |
| Lesions >25 mm^3^ | 370 (51%) | 266 (67%) | 636 (57%) |
| IVH | 146 (20%) | 51 (13%) | 197 (18%) |
| Cisterns compressed | 400 (56%) | 231 (58%) | 631 (57%) |
| Cisterns obliterated | 58 (8%) | 33 (8%) | 91 (8%) |
| **Rotterdam CT components** | |  |  |
| Cisterns compressed | 400 (56%) | 231 (58%) | 631 (57%) |
| Cisterns obliterated | 58 (8%) | 33 (8%) | 91 (8%) |
| EDH | 104 (14%) | 44 (11%) | 148 (13%) |
| IVH/SAH | 591 (82%) | 306 (77%) | 897 (80%) |
| Midline shift ≥5 mm | 278 (39%) | 177 (45%) | 455 (41%) |
| **Stockholm CT components** | |  |  |
| SAH-IVH | 146 (20%) | 51 (13%) | 197 (18%) |
| SAH-convexity 1-5mm | 450 (65%) | 224 (57%) | 674 (60%) |
| SAH-convexity >5mm | 99 (14%) | 57 (14%) | 156 (14%) |
| SAH-cisterns 1-5mm | 157 (22%) | 102 (26%) | 259 (23%) |
| SAH-cisterns >5mm | 46 (6%) | 22 (6%) | 68 (6%) |
| SAH-Score 0 | 129 (18%) | 89 (23%) | 218 (20%) |
| SAH-Score 1 | 269 (37%) | 146 (37%) | 415 (37%) |
| SAH-Score 2 | 142 (20%) | 64 (16%) | 206 (18%) |
| SAH-Score 3 | 111 (15%) | 54 (14%) | 165 (15%) |
| SAH-Score 4 | 46 (6%) | 28 (7%) | 74 (7%) |
| SAH-Score 5 | 19 (3%) | 10 (3%) | 29 (3%) |
| SAH-Score 6 | 4 (1%) | 5 (1%) | 9 (1%) |
| DAI on CT | 41 (6%) | 13 (3%) | 54 (5%) |
| No midline shift | 382 (53%) | 178 (45%) | 560 (50%) |
| Midline shift, mm (IQR) | 9 (5-14) | 10 (6-15) | 9 (5-14) |
| EDH | 104 (14%) | 44 (11%) | 148 (13%) |
| Dual ASDH | 56 (8%) | 20 (5%) | 76 (7%) |

S1 Table – Prevalence of CT components between centers

Prevalence of the components of investigated CT scores. Data is given as count and percentage of the patients combined and by center. However, midline shift, as in in millimeters is presented by the median and interquartile range. IQR = Interquartile range, ASDH = Acute subdural hematoma, ICH = Intracerebral hematoma, EDH = Epidural hematoma , IVH = Intraventricular hematoma, SAH = Subarachnoid hemorrhage, DAI = Diffuse axonal injury.
