# Supplementary material for: Evaluation of novel computerized tomography scoring systems in human traumatic brain injury: An observational, multicenter study
Source: PLoS Med. 2017 Aug 3;14(8):e1002368. doi: 10.1371/journal.pmed.1002368 (PMC5542385; doi:10.1371/journal.pmed.1002368)
Supplement: S2 Table — (DOCX) [file pmed.1002368.s003.docx]

| **S2 Table:** Pseudo-explained variance (Nagelkerke´s pseudo R^2^) for the subcomponents of the Base model and CT models | | | |
| --- | --- | --- | --- |
|  | **Stockholm** (n=720) | **Helsinki** (n=395) | **Combined** (n=1115) |
| **Base components** | | | |
| Age | 0.13 | 0.20 | 0.14 |
| GCS | 0.13 | 0.11 | 0.12 |
| Pupil responsiveness | 0.11 | 0.14 | 0.12 |
| Admission hemoglobin | 0.03 | 0.06 | 0.04 |
| Admission glucose | 0.06 | 0.15 | 0.10 |
| **Helsinki CT components** | | | |
| SDH | 0.03 | 0.07 | 0.04 |
| ICH/Contusions | 0.02 | 0.01 | 0.01 |
| EDH | 0.08 | 0.04 | 0.07 |
| Lesions >25 mm^3^ | 0.01 | 0.11 | 0.03 |
| IVH | 0.06 | 0.11 | 0.08 |
| Cistern compression | 0.05 | 0.15 | 0.08 |
| **Rotterdam CT components** | | | |
| Cistern compression | 0.05 | 0.15 | 0.08 |
| EDH | 0.08 | 0.04 | 0.07 |
| IVH/SAH | 0.04 | 0.04 | 0.04 |
| Midline shift ≥5 mm | 0.02 | 0.13 | 0.04 |
| **Stockholm CT components** | |  |  |
| SAH-IVH | 0.06 | 0.11 | 0.08 |
| SAH-convexity | 0.07 | 0.08 | 0.08 |
| SAH-cisterns | 0.04 | 0.06 | 0.05 |
| SAH-Score (total) | 0.13 | 0.10 | 0.12 |
| DAI on CT | <0.01 | <0.01 | <0.01 |
| Midline shift | 0.04 | 0.20 | 0.08 |
| EDH | 0.08 | 0.04 | 0.07 |
| Dual SDH | 0.05 | 0.05 | 0.05 |
| Table illustrating the explanatory relation of the individual components, combined and by center, towards unfavorable outcome (GOS 1-3 vs 4-5) using univariate logistic regression of un-imputed data. Results are illustrated as Nagelkerke´s R^2^. | | | |
