# Supplementary material for: Evaluation of novel computerized tomography scoring systems in human traumatic brain injury: An observational, multicenter study
Source: PLoS Med. 2017 Aug 3;14(8):e1002368. doi: 10.1371/journal.pmed.1002368 (PMC5542385; doi:10.1371/journal.pmed.1002368)
Supplement: S1 Text — The study protocol used when designing the study. (DOCX) [file pmed.1002368.s004.docx]

**S1 Text: ANALYSIS PLAN**

**AIM**

The aim is to assess the outcome prediction accuracy of the summed-scores of the two novel computerized tomography (CT) scoring system in traumatic brain injury (TBI), namely Stockholm and Helsinki CT scores, and compare them with the scoring systems/injury assessment tools used today (Rotterdam CT score and Marshall CT classification). This will be done in different populations from different hospitals, thus will constitute an external validation. We also wanted to see if the systems provided additional information to known outcome predictors of TBI and which sub-components of the scores that showed best association with outcome.

**METHODS**

*Study Population*

“Significant” TBI was defined in our study as patients admitted directly to the neuro-intensive care unit (NICU) in Karolinska University Hospital and Helsinki University Hospital for an acute traumatic brain injury. We believe this better constitutes a severely injured cohort than the use of Glasgow Coma Scale which is highly erratic, and influence by a multitude of non-trauma related factors the first 24 hours following injury.

We will include patients ≥15 years old, which normally constitute as “adults” in trauma situations, and admitted between 2005-2014 in Stockholm and 2013-2014 in Helsinki, thus not part of the original scoring cohorts for neither Stockholm- nor Helsinki CT scores.

We will exclude patients with incomplete follow-up, penetrating brain injuries, missing CT scans and patients admitted to the NICU for other reasons than the acute brain injury.

*Long-term outcome assessment*

In Stockholm, all TBI patients are prospectively followed-up using questionnaires for determining Glasgow Outcome Scale (GOS) at approximately 12 months following injury. In Helsinki, patients are prospectively followed up through telephone conference or in out-patient clinics at approximately 6 months post injury. The GOS will be dichotomized to GOS1-3 (unfavorable) vs GOS4-5 (favorable), GOS1 (dead) vs GOS 2-5 (alive) and as well as compared between all steps of GOS in a proportional model.

*Analyses*

The statistical program R (version 3.3.1, R-project, Vienna, Austria) is used for the statistical analyses.

Univariate analysis is performed using a logarithmic regression models correlating the different CT scoring systems with different outcome dichotomizations (bivariate), or proportional odds models versus all stages of GOS. Multivariable analyses will be performed using other known IMPACT variables available at both sites.

In the primary analysis, we will assess the association between CT complete sum scores and different dichotomizations of outcome in both the Stockholm and the Helsinki cohort. For this, we will use a the univariate regression model previously described, illustrating the explained variance of the outcome prediction models using Nagelkerke’s Pseudo-R^2^ and Receiver Operating Characteristic Area Under the Curve (ROC-AUC).

In the second analysis, we wish to analyze how different subcomponents of the scores were associated with outcome. This will be done in a similar univariate regression approach using Nagelkerke’s Pseudo R^2^ to highlight the accuracy of each component in the outcome model. Here, we will also include other univariate parameters (age, pupil unresponsiveness, Glasgow Coma Scale, admission hemoglobin and admission glucose)

In a third analysis, we will analyze how much of additional outcome information is explained by the outcome predictions if known predictors of outcome (high age, pupil unresponsiveness, Glasgow Coma Scale, admission hemoglobin and admission glucose) are used as a base model. Complete data sets will derived via a multiple imputation using the MICA package in R. In this multivariable model, each CT score will be looked at individually and we will note the additional explained variance in Nagelkerke’s Pseudo-R^2^.

In the primary analysis, data will this be presented as AUC values with 95% confidence intervals and as Nagelkerke’s pseudo-R^2^. For the rest, Nagelkerke’s pseudo-R^2^ will be used exclusively. P-values <0.05 will be considered statistically significant.
